# A Massive Ewing Sarcoma of the Rib: A Case Report with Literature Review

**DOI:** 10.1155/2022/6921004

**Published:** 2022-05-14

**Authors:** Naseem Fahad, Dima Hamideh, Rana Zareef, Samir Akel

**Affiliations:** ^1^Department of Pediatrics and Adolescent Medicine, American University of Beirut Medical Center, Beirut, Lebanon; ^2^Department of Surgery, American University of Beirut Medical Center, Beirut, Lebanon

## Abstract

A 9-year-old boy with a 16 cm chest wall mass, presenting with progressive cough and exertional dyspnea, was finally diagnosed with Ewing sarcoma of the rib. Such massive tumors usually present with metastasis and carry a bad prognosis. Fortunately, we present here a successful treatment approach for Ewing sarcoma of the ribs, defeating the overwhelming obstacles commonly faced in chest wall tumors. Delays in diagnosis, misdiagnosis, difficulty with general anesthesia, opportunistic infections, disruptions in chemotherapy delivery, and debilitating chest wall deformities are all potential challenges that could complicate the course of treatment.

## 1. Introduction

Ewing sarcoma is the second most common primary bone malignancy in children and adolescents. Rib Ewing sarcoma accounts for 13.4% of the total cases. Approximately 20–25% of patients present with metastases at diagnosis and are often resistant to intensive therapy [1]. On many occasions, diagnosis might be missed or delayed [2].

## 2. Case Report

A 9-year-old male presented to the emergency department with a 10-day history of progressive dry cough, chest pain, fatigue, and exertional dyspnea. Symptoms started one month prior to presentation when the patient suffered progressively exacerbating chest pain and cough, which became more prominent in the supine position. The symptoms have been severely aggravated in the last 10 days. Otherwise, the patient denied any change in appetite, fever, weight loss, headache, gastrointestinal symptoms, or urinary problems.

Prior to his presentation to the emergency department at our institution, he was seen in another hospital where a chest X-ray showed an abnormal consolidation and pleural effusion in the right hemithorax. On presentation, he was afebrile (temperature 37.8°C), tachycardic (heart rate of 112 beats per minute), normotensive (126/76 mmHg), slightly tachypneic (respiratory rate of 27 breath/min), and saturating at 100% on room air.

The physical exam was remarkable for the use of accessory muscles for breathing and decreased breath sounds on the right upper, middle, and lower fields. In addition, a palpable mass measuring around 3 cm in diameter was noted on the anterior right chest, associated with right-sided axillary lymphadenopathy. No other pertinent positives were detected.

**Figure 1:**
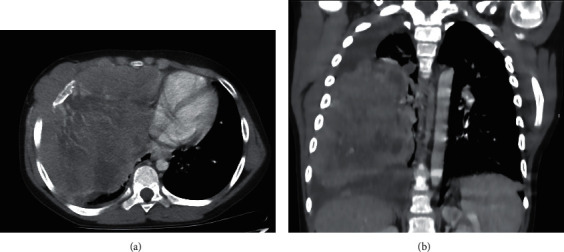
(a, b) A CT chest performed at the initial encounter. The CT chest obtained at the initial encounter revealed a large, heterogeneously enhancing mass occupying the majority of the right hemithorax with peripheral necrosis.

A CT chest with contrast was performed (Figures 1(a) and 1(b)) which showed a very large heterogeneously enhancing mass occupying the majority of the right hemithorax with peripheral necrosis measuring approximately 16 × 14 × 11 cm (craniocaudal by anteroposterior by transverse dimensions). No interval calcification was seen on the precontrast images. The mass invaded the right chest wall at the level of the lateral aspect of the fifth and sixth ribs on the right side, where it extended into the sella at the anterior muscle. Bone erosion and deformity of the right sixth rib and, to a lesser extent, of the inferior aspect of the fifth rib were also seen.

**Figure 2:**
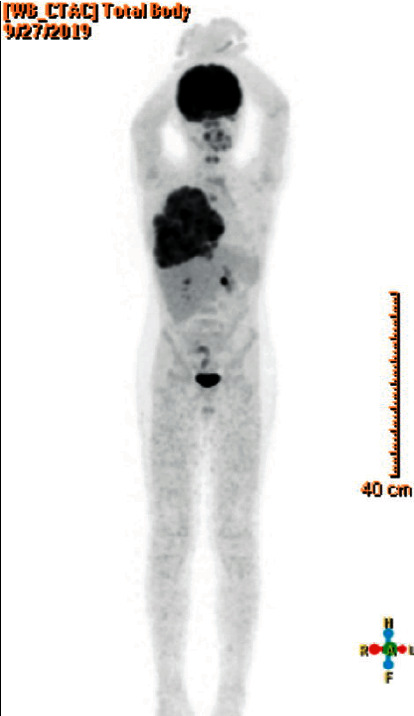
A PET scan performed at the initial encounter. The PET scan obtained before starting treatment showed a large soft tissue mass occupying the right hemithorax, invading the anterior chest wall and the overlying ribs, consistent with the known Ewing sarcoma. There was no evidence of FDG-avid disease in the rest of the body.

Based on the history, physical exam, and imaging results, the patient was admitted and a PET scan was performed (Figure 2). It revealed a large soft tissue mass occupying the right hemithorax and invading the anterior chest wall and the overlying ribs, which is consistent with Ewing sarcoma. No evidence of FDG-avid disease in the rest of the body was appreciated.

Thereafter, the decision was made to perform a CT-guided biopsy. The procedure was carried out under sedation, and 5 fragments of soft tissue measuring in aggregate 0.7 × 0.5 × 0.2 cm were submitted to the pathology lab.

Histopathology revealed a small-blue-round cell tumor. Immunostaining was remarkable for positive synaptophysin, CD99, and FLI-1. The PAS and PASD stains highlighted the cytoplasmic glycogen. CKAE1/3, chromogranin, desmin, S100, MYOD1, Myogenin, CD45, and TdT were all negative. The pathology result was consistent with Ewing sarcoma. Accordingly, he was diagnosed with Ewing sarcoma of the chest wall, with staging of local disease.

A solid tumor panel by molecular testing of RT-PCR was also taken, but the results could not be obtained urgently as they were not locally available and had to be sent to another country.

Initially, the patient was planned for bilateral bone marrow aspirate and biopsy, and the insertion of a central line (Polysite). However, they could not be carried out due to the difficulty of general anesthesia at that time. Instead, central access was secured through a peripherally inserted central catheter (PICC) to start chemotherapy after obtaining a baseline echocardiogram. The patient was initiated on CHILDREN'S ONCOLOGY GROUP protocol AEWS0031, which consists of a total of 43 weeks of treatment. Chemotherapy consists of 14 cycles of chemotherapy: 6 cycles of induction, followed by 8 cycles of consolidation after local control through surgical excision or radiotherapy. Each cycle is over 14 days in length with an alternation between vincristine/cyclophosphamide/doxorubicin/GCSF (VDC) and ifosfamide/etoposide/GCSF (IE).

The treatment course was complicated by a perianal abscess after cycle 1 of VDC, managed by drainage. The culture remained negative, but 16S from abscess fluid disclosed *Prevotella bivia*. The patient was treated with meropenem, vancomycin, and amikacin for a total of 14 days. The patient also developed severe dermatitis over the site of the PICC line, which required line removal and treatment with clobetasol (a local steroid). The patient had to receive his second cycle of chemotherapy IE through a peripheral line.

After completion of the second cycle, the solid tumor panel resulted from molecular testing of RT-PCR confirmed the diagnosis. The patient's tissue cell lysate was positive for t(11; 22) (21; 22) EWS/FLI-1/ERG fusion transcript.

A CT chest was obtained after the second cycle of chemotherapy, which showed a significant decrease (almost 50%) in the size of the enhancing pleural mass in the right hemithorax, with resolution of its mass effect on the adjacent cardio-mediastinal structures. The mass that was previously measuring 16 × 14 × 11 cm (CC *x* AP *x* TR) decreased to a size of 6.5 × 8.9 × 4.1 cm in its maximum dimensions.

The patient had a central venous access (Polysite) inserted under general anesthesia. A bone marrow biopsy and aspirate were obtained and were negative for metastatic disease.

**Figure 3:**
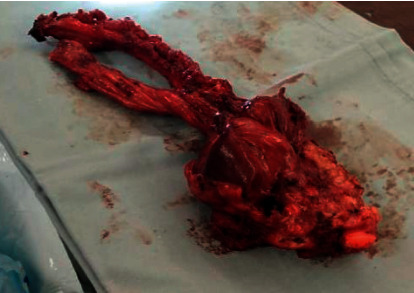
This is the en bloc resected specimen showing the involved ribs completely resected (on the left side) with the residual Ewing sarcoma tumorous mass, including the thoracic muscles (on the right side of the picture), resected to secure free margins.

The patient continued to receive the planned 6 cycles of induction chemotherapy, then local control by surgical resection was decided. The surgery involved the resection of part of the 5th rib, total resection of the 6th rib, right lower lobe wedge resection, and partial resection of the right middle lobe. In addition, two other pulmonary nodules were detected in the right middle lobe and were removed. Segmental lung resection had to be performed on the stuck right lower lobe, with pleurectomy as an en bloc with the whole specimen (Figure 3). No complications were encountered during surgery.

On gross examination, the tumor was exophytic in nature, going intrathoracically and definitely infiltrating the major fissure between the right middle lobe and right lower lobe and also stuck to the diaphragm. The tumor pathology revealed fibrosis and granulation tissue, which was consistent with the treatment effect. No viable residual tumor tissue was identified in the specimen or the lymph nodes.

As the patient had an R0 resection with a complete response, and as per the recommendations, there was no indication for adjuvant radiotherapy. However, since he initially had a large lesion, with suspicious pleura involvement that was not sampled, and due to its location involving the chest wall, radiation therapy was opted for.

The patient was started on radiotherapy 2 weeks following surgery and after securing proper healing of the chest wall scar. The patient received radiation for the whole involved lung with 15 Gy for 10 sessions, followed by a boost to the surgical bed to a dose of 50.4 in 1.8 Gy per fraction.

The patient later continued his chemotherapy plan for a total of 43 weeks (14 cycles of chemotherapy).

The CT chest and PET scan at the end of treatment confirmed complete remission. Currently, the patient has been in remission for more than one year without a relapse. The patient is being regularly followed up with an imaging evaluation every 3-4 months.

## 3. Discussion

Ewing sarcoma is the most common malignant chest wall tumor in children and adolescents. Indeed, more than 50% of the chest wall tumors are chondrosarcoma and Ewing sarcoma [3]. Clinically, the presentation of these tumors widely varies and can include pain, shortness of breath, chest wall mass, among others. In rib Ewing sarcoma, pain is the most common reported presentation. Besides, around 50% of cases present with a chest wall mass, which should raise suspicions about Ewing sarcoma [2]. It can be sporadic or secondary to previous treatment, such as leukemia [4].

The diagnosis of Ewing sarcoma among the chest wall tumors remains a challenging task. In addition, the dilemma of initiating the proper treatment can be time-consuming. Pleurisy, rib fracture, or muscle strain are the most common misdiagnosis of rib Ewing sarcoma [2]. Misdiagnosis of tuberculosis can be often faced as well [5]. The accurate diagnosis is always based on pathologic findings associated with immunohistochemistry studies and molecular testing [6].

Initiating chemotherapy before approaching local control with surgery or radiation is advisable. Indeed, delaying surgery is found to be correlated with higher rates of successful complete resection of the Ewing sarcoma [7]. The decision for surgical intervention is usually endorsed after chemotherapy, in order to have a better assessment of tumor limits. The definition of safe margins is variable and can range from 20 mm to 40 mm [8, 9]. R0 resection of the tumor can be attained in many experienced centers, with resection of the involved rib reaching up to 100%. Whereas, R1 patients have to undergo another resection, if possible, for a better prognosis [9]. Another well-grounded treatment option is adopting high-dose chemotherapy followed by a stem cell transplant [10].

Several factors might complicate the course of treatment. Chemotherapy resistance is a major obstacle and is usually encountered with large tumors or in specific patients profiles [11]. Moreover, chemotherapy delays with a high frequency of interruptions during treatment might reduce overall survival [12]. Unfortunately, over 70% of patients experience interruptions in chemotherapy due to central line infection [13]. Besides, 60% of cases exhibit pleural effusion at the time of diagnosis, which is usually associated with a poor prognosis [14].

A surgical approach with thoracoscopic guidance is another modality to avoid a lateral thoracotomy, thereby enhancing postoperative recovery and reducing pain. Resection of the anterior or lateral chest wall with more than 3 ribs is considered a critical choice as it might lead to a flail chest deformity and acute respiratory failure [15].

In this case, the persistence of chest pain and the large mass were alarming signs, which were overlooked initially. Fortunately, he only exhibited localized disease without metastasis at presentation. A high index of suspicion and the prompt initiation of chemotherapy have been key aspects of his prognosis. Chest wall tumors commonly encounter difficulties in keeping the patient under general anesthesia and securing central access. Delivering chemotherapy through a peripherally inserted intravenous line was used to avoid delays. Abating the tumor size with chemotherapy cycles separated by 14 rather than 21 days hastened the reduction of the tumor size and facilitated R0 resection with fewer debilitating surgical consequences.
